# Resveratrol Analogues as Selective Estrogen Signaling Pathway Modulators: Structure–Activity Relationship

**DOI:** 10.3390/molecules27206973

**Published:** 2022-10-17

**Authors:** Paulina Kobylka, Malgorzata Kucinska, Jacek Kujawski, Dawid Lazewski, Marcin Wierzchowski, Marek Murias

**Affiliations:** 1 Department of Toxicology, Poznan University of Medical Sciences, 60-631 Poznan, Poland; 2 Chair and Department of Organic Chemistry, Poznan University of Medical Sciences, 60-780 Poznan, Poland; 3 Chair and Department of Chemical Technology of Drugs, Poznan University of Medical Sciences, 60-780 Poznan, Poland

**Keywords:** resveratrol, estrogen receptor, resveratrol analogues

## Abstract

Resveratrol is a plant-derived phytoalexin found in grapes, red wine and many other plants used in Asian folk medicine. It is extensively studied for pleiotropic biological activity. The most crucial are anticancer and chemopreventive properties. Resveratrol has also been reported to be an antioxidant and phytoestrogen. The phytoestrogenic activity of resveratrol was assayed in different in vitro and in vivo models. Although these works brought some, on the first look, conflicting results, it is commonly accepted that resveratrol interacts with estrogen receptors and functions as a mixed agonist/antagonist. It is widely accepted that the hydroxyl groups are crucial for resveratrol’s cytotoxic and antioxidative activity and are responsible for binding estrogen receptors. In this work, we assayed 11 resveratrol analogues, seven barring methoxy groups and six hydroxylated analogues in different combinations at positions 3, 4, 5 and 3′,4′,5′. For this purpose, recombined estrogen receptors and estrogen-dependent MCF-7 and Ishikawa cells were used. Our study was supported by in silico docking studies. We have shown that, resveratrol and 3,4,4′5′-tetrahydroxystilbene, 3,3′,4,5,5′-pentahydroxystilbene and 3,3′,4,4′,5,5′-hexahydroxystilbene may act as selective estrogen receptor modulators.

## 1. Introduction

Resveratrol is a plant-derived phytoalexin found in grapes, red wine and many other plants used in Asian folk medicine [1]. It is extensively studied for pleiotropic biological activity. As the most crucial, its anticancer and chemopreventive properties may be mentioned [1]. Resveratrol has also been reported to be an antioxidant and phytoestrogen [2,3,4,5]. At the molecular level, its biological and antioxidant activity is mediated by the presence of hydroxyl groups. Their distribution in aromatic rings in the stilbene scaffold makes it similar in chemical structure to endogenous and synthetic estrogens such as 17β-estradiol (E2) or diethylstilbestrol [3]. The phytoestrogenic activity of resveratrol was assayed in different in vitro and in vivo models [3], including estrogen receptor-expressing breast, ovarian and endometrial cancer cells, as well as luciferase reporter gene transfected cells [6,7,8]. Although these works brought some, on the first look, conflicting results, it is commonly accepted that resveratrol interacts with estrogen receptors (ERs) and functions as a mixed agonist/antagonist [9,10,11,12]. The activity of resveratrol was most fully known in the case of breast cancer, in which its influence on cell proliferation, metastasis, epigenetic alterations, induction of apoptosis and sensitization toward chemotherapeutic drugs has been evaluated in various in in vitro and in vivo models [13]. It should also be mentioned that resveratrol also interferes with estrogens’ intestinal and hepatic metabolism, including its impact on steroidogenesis (CYP17A1, CYP19 and CYP21A1) and biotransformation of estrogens and steroids (CYP1A1, CYP1A2, CYP1B1, UGTs, SULT1A1 and SULT1E1). That may significantly change their levels and modulate their central and peripheral actions; their interaction with ERs seems to play a crucial role [3]. The theoretical basics of their interactions with estrogen receptors were described in works presenting its in silico docking to estrogen receptors [6,14]. The hydroxyl groups are similarly responsible for binding estrogen receptors as though they are crucial for resveratrol’s cytotoxic and antioxidative activity [15]. The relationship between the pro-/antioxidant properties and cytotoxicity of higher resveratrol analogues (possessing more than three phenolic groups) has been explained in our previous work [2]. In this study, resveratrol analogues with pyrogallol and resorcinol moieties were used. The oxidation of ortho-hydroxystilbenes in our experimental system resulted in the production of ortho-semiquinones, which, in living cells, undergo redox cycling, thereby consuming additional oxygen and forming cytotoxic oxygen radicals. In contrast to compounds with other substitution patterns, hydroxystilbenes with one or two resorcinol groups (e.g., resveratrol) did not show an additional oxygen consumption or semiquinone formation. These findings suggest that ortho-semiquinone production mediates the increased cytotoxicity of ortho-hydroxystilbenes during metabolism or autoxidation. It makes resveratrol analogues attractive anticancer agents. As previously postulated, resveratrol and some of its analogues may stimulate the proliferation of cancer cells via both estrogenic and hormetic mechanisms [16]. In this study, we focused on the ability of resveratrol analogues to bind estrogen receptors and, consequently, the relationship between the estrogenic and cytotoxic activity of the resveratrol analogues.

## 2. Results and Discussion

Natural products are still at the center of attention due to their wide range of biological and therapeutic effects. At the same time, a significant percentage of drugs registered yearly are modifications of natural compounds [17]. Most of them are antibacterial and anticancer agents. In this study, we used in vitro and in silico techniques to investigate the activity of resveratrol analogues to modulate the activity of estrogen receptors. Currently, it is recognized that two receptors, ERα and ERβ, mediate the effects of estrogens. Both receptors are involved in several physiological and pathological processes [18]. It may also be indicated that ERα and ERβ regulate processes connected with the female reproductive system in regulating several other physiological and pathophysiological processes in the human body. Disrupted ER signaling leads to the development of different diseases, such as osteoporosis, neurodegeneration, inflammation and metabolic and cardiovascular diseases [19]; however, the most important indications to modify their activity connected with estrogen-related cancers such as breast [19], endometrial [20], cervical [21] and ovarian cancer [22].

In breast cancer, the presence of the ERα correlates with a better prognosis and a higher likelihood of a response to therapy. This is because more than half of breast cancers are ERα overexpressing, and about 70% of them respond to antiestrogen therapy (for example, tamoxifen). On the other hand, an increased expression of ERα and ERβ in benign breast epithelium seems to indicate an increased risk of breast cancer, suggesting a role for ERα in breast cancer initiation and progression [19,21].

The role of ERβ and its variants in cancerogenesis, breast cancer progression and prognosis response to therapy is even more complex and not fully understood despite it being over 25 years since its discovery. It seems evident that understanding is crucial for the further design of diagnostic and therapeutic strategies not only for breast and endometrial cancer [20,23]. Although we know relatively the most about the effect of ERβ on these cancers, the situation seems much more complicated than in the case of ERα. There are theories that ERβ may play an ambivalent role in breast cancer, activating different pathways and functions in the presence and absence of ERα [18,23]. It was postulated that ERβ exerts an antiproliferative effect in breast cancer cells in the presence of ERα but has a proliferative impact in the absence of ERα [24].

The interaction of resveratrol with estrogen receptors and its impact on estrogen-responsive cancer cells has been presented in several works that brought up some conflicting results [10,12,13,25]. Resveratrol acts as a mixed agonist/antagonist for ERα and ERβ. All available data has been summarized recently by the review work prepared by Qasem [3]. In this paper, we move forward and test resveratrol analogues in silico and in vitro using recombinant estrogen receptors and estrogen-dependent MCF-7 (breast-derived) and Ishikawa (endometrial) cancer cells.

### 2.1. Binding Affinity of Resveratrol and Its Analogues to ERα and ERβ

**Figure 1:**
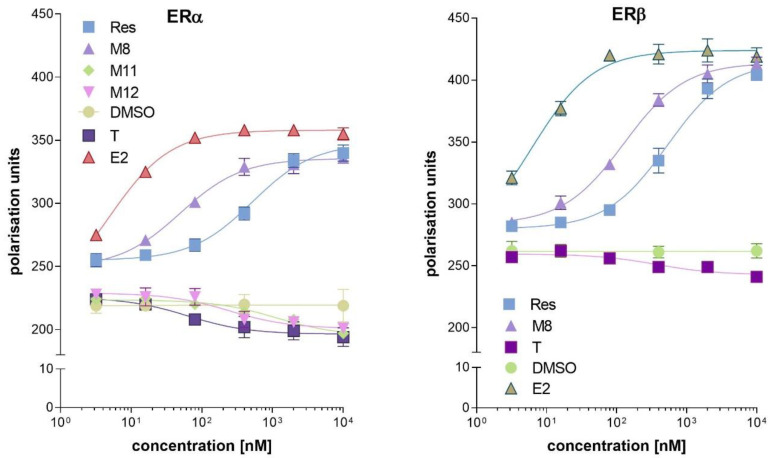
Interaction of resveratrol analogues with human estrogen receptors: ERα (left panel) and ERβ (right panel). In this experiment, only the active compounds are shown. Abbreviations used: Res—resveratrol, **M8**—3,4,4′,5-tetrahydroxystilbene, **M11**—3,3′4,5,5′-pentametoxystilbene, **M12**—3,3′,4,4′,5,5′-hexahydroxystilbene, T—tamoxifen, E2—estradiol as a solvent and control DMSO—dimethyl sulfoxide were used.

The binding of a ligand to the ER is crucial for its potential to act as an ER agonist or antagonist. In this assay, we tested resveratrol and its 11 analogues shown in Table 1. The EC_50_ value of the ligands that either promote or disrupt the ER/D22 interaction provided a means to classify the test compound as an antagonist or agonist. Of the tested compounds, only **M4** showed an interaction with recombinant estrogen receptors. The agonistic activity for ERα of resveratrol and compound **M8** was shown. Compounds **M11** and **M12** exhibited antagonistic activity for ERα. Resveratrol and compound **M8** could act as ERβ agonists, while compound **M12** did not show an antagonistic effect in ERβ (Figure 1). Based on a fluorescence polarisation assay with recombinant estrogen receptors, four compounds (resveratrol, **M8**, **M11** and **M12**) were selected for further tests (Table 2).

### 2.2. Docking Studies

**Figure 2:**
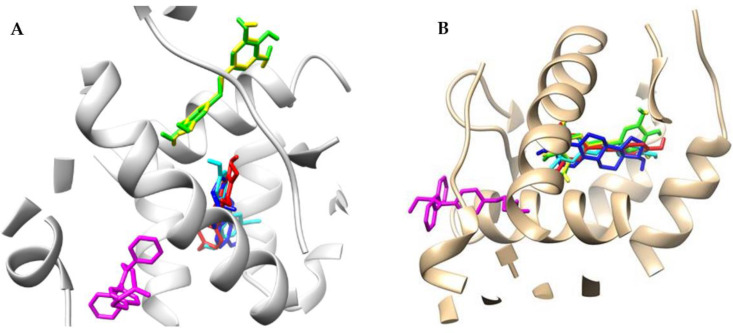
Superimposition of docked ligands: **M8** (red), **M12** (green), **M11** (yellow), resveratrol (cyan), estradiol (blue) and tamoxifen (magenta); first poses (Chimera 1.13.1 package) protein (contacts under d ≤ 9 Å) in estrogen receptor α (A; 3dt3.pdb) and estrogen receptor β (1l2j.pdb). Panel **A** presents ERα while panel **B** shows ERβ.

**Figure 3:**
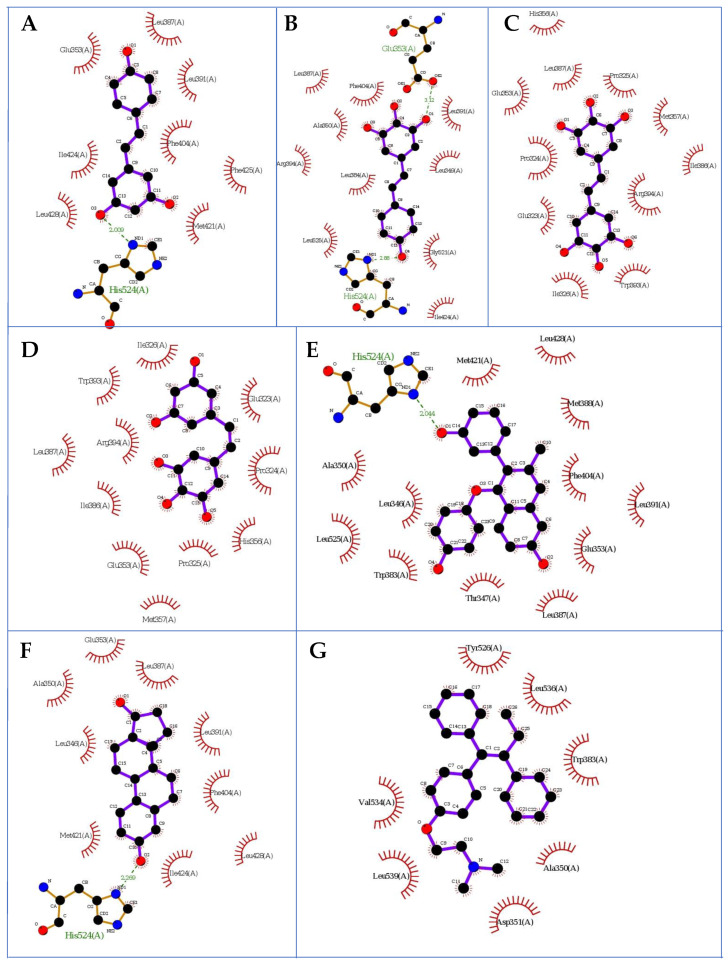
The first poses of the docked ligands to ERα: (**A**) resveratrol, (**B**) **M8**, (**C**) **M12**, (**D**) **M11**, (**E**) tamoxifen, (**F**) estradiol and (**G**) GW368; hydrogen atoms are omitted; 3dt3.pdb protein (LigPlot+ v.2.2 software).

**Figure 4:**
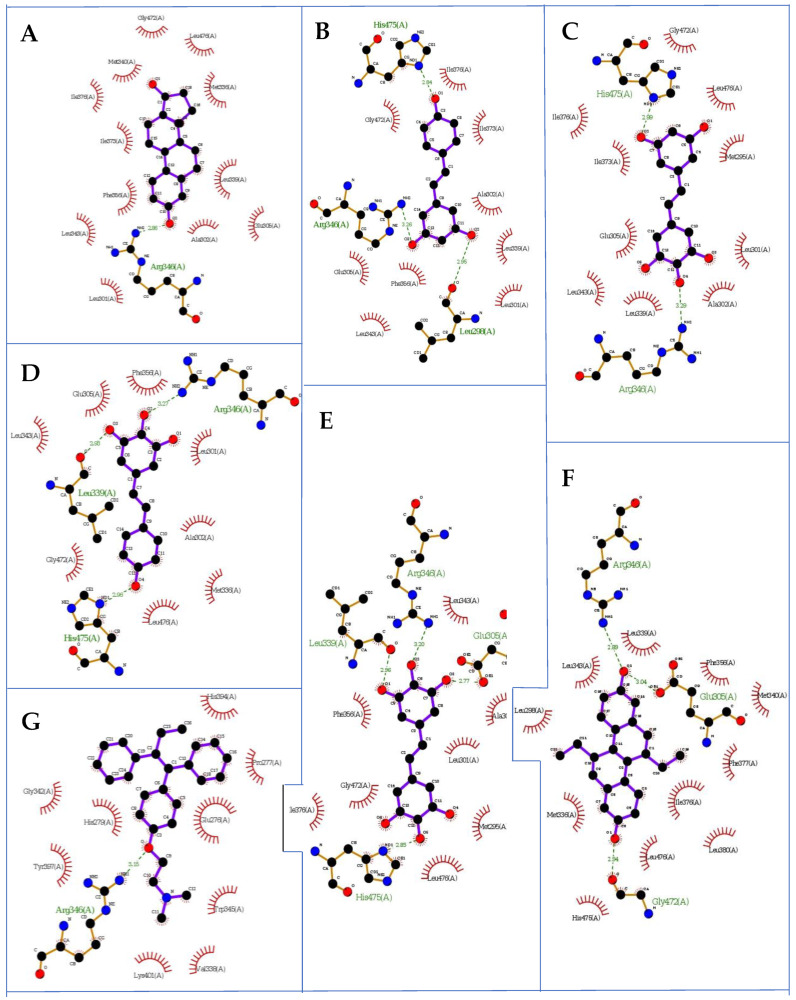
The first poses of the docked ligands to ERβ: (**A**) estradiol, (**B**) resveratrol, (**C**) **M11**, (**D**) **M8**, (**E**) **M12**, (**G**) tamoxifen, and (**F**) GW368; hydrogen atoms are omitted; 3dt3.pdb protein (LigPlot+ v.2.2 software).

Fitting the first poses of compounds **M8**, **M12**, **M11**, resveratrol (in the E conformation), estradiol and tamoxifen in the human estrogen receptor ERα domain by using protein 3dt3.pdb [26,27] that contained a GW368 ligand resulted in forming several hydrogen bonds between the ligands and the protein amino acids. In the docking procedure, we considered a distance d ≤ 4.0 Å between a proton and a heteroatom of the adjacent molecule (Table 3, Figure 2 and Figure 4). We observed that the binding modes of the docked conformers (first poses, Figure 2) of ligands **M8**, estradiol and resveratrol were almost identical. Moreover, considering the overlap of the docked **M12** and **M11** ligands, we observed that their distance from **M8** alkene was ca. 4 Å. The tamoxifen, however, was positioned differently at a distance of ca. 9 Å from the **M8**, **M12** and **M11** systems. This binding pattern had particular importance for the interactions involving the polar OH groups in the pocket. Thus, in the protein pocket, we observed different distributions of functional groups of ligands **M8**, **M12** and **M11** in comparison with resveratrol, estradiol and tamoxifen. The estimates during the docking protocol binding affinity were as follows: −6.800, 6.900, −7.000, −7.600, −7.900 and −9.00 kcal mol^−1^ for: **M11**, tamoxifen, **M12**, **M8**, resveratrol and estradiol, respectively. Based on the literature data, the most interacting amino acid residues surrounding this cavity were selected, i.e., Leu346, Glu353, Trp383, Leu387, Phe404 and His524, to name a few [28,29]. On this account, we observed the O…H-N type of hydrogen bond formed between the hydroxyl functionality of particular ligands (its length was: 2.046, 2.009 and 2.269 Å for **M8**, resveratrol and estradiol, respectively; Figure 3) and protonated nitrogen atom within His524. We reported the same type of contact in our previous study [28] as it was related with the resveratrol and fused aromatic compound GW2368.

Our previous findings led us to draw the assumption that tryptophan present at position 383 is considered as a conservative point in the hormone binding site, and it is also present in other steroids receptors, probably due to its longest hydrophobic chain among natural amino acids [18]. Thus, Trp383 can interact with the hydrophobic region of estradiol. From this standpoint, herein, we decided to analyze the interactions of docked alkenes with Trp383. The distance of the pyrrolic N-H atom of this amino acid and the double bond of tamoxifen or the carbonyl group of Trp383 and hydroxyl moiety within the docked resveratrol equalled ca. 5 or 6 Å, respectively. Moreover, the distance between the N-H nitrogen atom of Trp383 and methylene protons within the structure of tamoxifen equalled ca. 5.5 Å. Since the **M8** ligand was located similarly to resveratrol, the observed contacts between this alkene and Trp383 were comparable to resveratrol. Despite the distance between the carbonyl group of Trp383 and hydroxyl functionality within the structure of the docked **M12** and **M11** ligands being equalled ca. 6 Å, their phenolic rings were almost perpendicular in comparison with the plane of the tryptophan’s indole system and carbonyl moiety. Notably, the phenyl rings of Trp383 and tamoxifen (distance equalled ca. 4 Å) and **M8** ligand (distance equalled ca. 6 Å) were coplanar.

A typical O-H…OOC hydrogen bond was formed by the interaction of the hydroxyl group of **M8** and carboxyl functionality of Glu353 and equalled 3.120 Å (Figure 3). These types of contacts, however, exceeded the distance of 4 Å in the cases of **M12** and **M11** alkenes. We observed them in our previous study as the internal GW2368 ligand formed them within the 3dt3.pdb complex [28].

Our computations allowed us to observe that the distance between the carbonyl group of Leu346 and the hydroxyl functionality of **M12** and **M11** equalled 3.143 and 2.710 Å, respectively; however, the rotated (axial) position of the hydroxyl group within the **M12** make this contact significantly weaker in comparison with the **M11** analogue. The same type of contact was observed concerning Leu387 and **M8** (distance equalled 3.425 Å). The distance between the amine group of Leu387 and hydroxyl moiety of **M11** equalled 3.193 Å. Still, it led us to conclude that the contacts of the mentioned ligands with both Leu346 and Leu387 seemed significantly weak.

Weak contacts of the analyzed alkenes were detected regarding the possible interactions of the **M8**, **M12** and **M11** ligands with Phe404, which was nearly neglected in our previous study [28]. Considering the possible formation of π–π stacking and C=O…H-O or C-H…O-H contacts, we concluded that the closed location to Phe404 was noticed within the **M11** 3dt3 complex.

Next, we employed the above data to analyze the interaction energy of ligands **M8**, **M12** and **M11** with the neutral amino acids involved in the hydrogen bonding or π–π stackings (Table 3). For this purpose, we used the SAPT (symmetry-adapted perturbation theory) approach, which provides the interaction energy’s decomposition into physical components: i.e., electrostatic, exchange, induction and dispersion terms. A detailed description of this protocol is described in our previous investigations [30]. For the interactions with Glu353, the lowest (the most negative value) total energy SAPT0 was calculated for estradiol (−15.11974 kcal mol^−1^) and **M12** (−13.62578 kcal mol^−1^); however, its value computed for **M8** (15.21372 kcal mol^−1^) was not favorable. It turned out that, energetically, the diffuse charge of the amino acid’s carboxylic group did have an important influence on the O-H…OOC type of contact. The same analysis by the SAPT approach showed that the interaction energy involving Leu346 was the lowest for estradiol (−7.61105 kcal mol−1) and resveratrol (−3.20152 kcal mol^−1^); however, contacts of other analyzed ligands with Leu346 turned out to be significantly weakened. The interactions of the discussed ligands were also observed considering Leu387 and **M11** and **M12** derivatives. The lowest total energy SAPT0 was calculated for resveratrol (−4.45619 kcal mol^−1^) and **M8** (−2.86244 kcal mol^−1^). On the other hand, the interactions involving Phe404 had the most negative value in the presence of ligands: **M8** (−6.75066 kcal mol^−1^) and resveratrol (−2.9597 kcal mol^−1^). It seemed to originate due to dispersion (−5.83775 and −7.92448 kcal mol^−1^ for **M8** and resveratrol, respectively) and electrostatic terms (−1.37589 and −2.67406 kcal mol^−1^ for **M8** and resveratrol, respectively). For the interactions with Trp383, the lowest total energy SAPT0 was calculated for tamoxifen (−5.57119 kcal mol^−1^). For interactions within the Trp383 **M8** and Trp383 **M11** complexes, however, we detected that the total SAPT0 energy equalled: −1.21807 and −1.26194 kcal mol^−1^, respectively, which was comparable with energy suitable complex formed by estradiol (−1.21379 kcal mol^−1^) and resveratrol (−1.0303 kcal mol^−1^). Finally, the exchange nature of the energetic term was not detected during the analysis of the analyzed Leu346 **M11**, Leu346 **M12** and Phe404 tamoxifen complexes using the SAPT method. The above findings support the conclusions drawn from the docking studies. The results of our investigations, involving the multilevel approach, confirm the presence of interactions between the alkene ligands **M8**, **M12** and **M11** and amino acids of the ERα pocket, especially involving: Glu353, Trp383 and Phe404.

As a result of computations related to the receptor ERβ (1l2j.pdb taken from the Protein Data Bank database with the ETC compound as an internal ligand (‘Human ERβ Ligand-binding Domain in Complex with (*R*,*R*)-5,11-cis-diethyl-5,6,11,12-tetrahydrochrysene-2,8-diol), we also observed that the binding modes of the docked conformers (first poses, Figure 3) of ligands **M8**, **M12**, **M11**, estradiol and resveratrol were similar. The estimates during the docking protocol binding affinity were as follows: −9.500, −8.00, −7.900, −7.700, −7.600 and −7.000 kcal mol^−1^ for: estradiol, resveratrol, **M8**, **M12**, **M11** and tamoxifen, respectively. Based on the literature data, the most interacting amino acid residues surrounding this cavity were selected, i.e., Leu298, Met336, Met340, Leu339, Met340, Leu343, Arg346, Ile376, Ile380, Gly472 and His475, to name a few [31,32]. For the interactions with Leu298, Met336, Leu339, Met340, Leu343, Ile376, Ile380, Gly472 and His475, respectively, the lowest (the most negative value) total energy for SAPT0 was calculated as (Table 4): **M11** (−9.13157 kcal mol^−1^), resveratrol (−3.6376 kcal mol^−1^), **M12** (−13.64053 kcal mol^−1^), resveratrol (−4.01457 kcal mol^−1^), **M8** (−2.06224 kcal mol^−1^), **M11** (−9.13157 kcal mol^−1^), resveratrol (−3.6376 kcal mol^−1^), **M12** (−13.64053 kcal mol^−1^), resveratrol (−4.01457 kcal mol^−1^), **M8** (−2.06224 kcal mol^−1^), **M11** (2.24053 kcal mol^−1^), **M8** (−0.29007 kcal mol^−1^), **M11** (−1.38151 kcal mol^−1^) and **M11** (−11.7796 kcal mol^−1^), respectively, for which basically the electrostatic nature of these contacts is responsible.

It is noteworthy that the computed interaction energy of **M8** and **M12** with Met336 is at a comparable level within the complex with particular amino acids. An analogous assumption might be drawn regarding the relations between **M8** and M 11 and Leu343 or Ile380. Finally, during the analysis of all tamoxifen amino acid complexes using the SAPT method, the exchange nature of energetic terms was not detected concerning: Gly472, His475, Ile376, Ile380 and Leu298 (Figure 4).

The resulting data regarding the ERα receptor (3dt3.pdb protein) were in agreement with our previous study [26]. They led to the conclusion that, for interactions, the alkene ligands **M11** and **M12** with amino acids within the ERα cavity were especially involved: Glu353, Trp383 and Phe404. Moreover, according to data from the docking protocol and the SAPT analysis, we can assume that stilbenes **M11** and **M12** showed antagonistic activity towards the ERα receptor. On the other hand, the docking poses of the **M8** and **M12** ligands within the cavity of the ERβ receptor (1l2j.pdb protein) were quite similar, and the computed interaction energies of these alkenes were at a comparable level. Moreover, the performed SAPT analysis proved that the interactions of the **M8** and **M12** derivatives with amino acids within the ERβ cavity were crucial Met336 and Leu343, and these amino acids turned out to be most interactive within the ERβ protein [29,30]. Thus, our computations proved the agonistic activity of the **M8** analogue towards ERα and ERβ.

### 2.3. Impact on the Proliferation of Estrogen-Dependent MCF−7 and Ishikawa Cell Lines—In Vitro Study

The relative estrogenic activity of resveratrol and its analogues were assayed in vitro using two estrogen-dependent cancer cell lines. Their response to estrogen stimulation in the alkaline phosphatase assay was used in well-differentiated endometrial Ishikawa cells, while a fluorescence-based assay was used to assess the proliferation of epithelial breast adenocarcinoma-MCF-7 cells. Both cell lines, Ishikawa and MCF-7, express estrogen receptors and are sensitive to estrogen treatment. Their detailed characteristics, including the expression of ERs at the mRNA and protein levels, were described previously in works dedicated to endometrial [33,34,35,36] and breast [37,38,39] cancer cell lines. In our work, we used concentrations ranging from 10 to 10,000 nm (0.01 to 10 µM), which covers the serum concentration of free resveratrol reported in diverse studies evaluating the bioavailability of resveratrol from different products and various matrices [40], e.g., white wine containing resveratrol 25 mg applied for healthy volunteers and resulted in C_max_ = 2.1 µM (480 µg/L) at T_max_ = 0.6 h [41], tablets 500 mg resulted in C_max_ = 311 nM.

**Figure 5:**
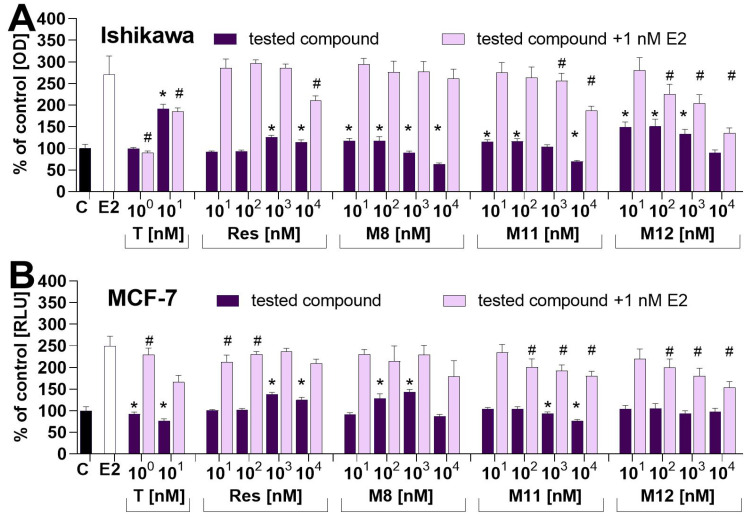
Dose–response of tested estrogen-dependent cancer cells. Cells were stimulated with: 1 nM estradiol (bar), tested compounds (light violet bars) and tested compounds incubated with 1 nM estradiol (dark violet bars) for 72 h. The control is illustrated by the black bar. (**A**) Proliferation of Ishikawa cells was measured by alkaline phosphatase activity. (**B**) Results obtained for MCF-7 cells; their proliferation was evaluated by fluorescence-based DNA measurements. The asterisks show a statistically significant difference between the control cells and cells stimulated by tested compounds: * *p* < 0.05. The pound symbol shows a statistically significant difference between cells incubated with 1 nM estradiol and cells incubated with 1 nM estradiol and tested compounds: # *p* < 0.05.

Some (71.1 ng/mL) at T_max_ = 1339 h [42] up to 5 g resulted in C_max_ = 2.57 µM (538.8 ng/mL) at T_max_ = 1.5 h [43]. In both cell lines, resveratrol at concentrations 10^3–^10^4^ nM stimulated an increase in the level of the assessed markers. Compounds **M8**, **M11** and **M12** stimulated the alkaline phosphatase in Ishikawa cells at 10–100 nM concentrations. In contrast, the activity of this marker enzyme in Ishikawa cells incubated with compounds **M8**, **M11** and **M12** at concentrations of 10^3^–10^4^ nM was significantly lower compared with the control. In MCF-7 cells incubated with **M8**, an increased DNA synthesis was detected in cells incubated with this compound at concentrations 10^3^–10^4^ nM, while. in MCF-7 cells incubated with **M11** at concentrations 10^3^–10^4^ nM, the DNA level was significantly lower compared with the control (Figure 5). In the experiment in which the tested compounds were incubated with E2, only the highest concentrations significantly changed the proliferation of Ishikawa and MCF-7 cells (Figure 5). The impact of resveratrol on the proliferation of different estrogen-dependent [10,44,45] and estrogen-independent cells transfected with estrogen receptors [12,45,46], as well luciferase reporter genes [10,35,47], has been assayed in past years in several papers delivering, in some cases, conflicting results. The impact of resveratrol has also been assayed in experiments where estrogen receptor-dependent cells were incubated with resveratrol and estradiol in Ishikawa cells [35] and MCF-7 [12,46].

From the beginning of research on the estrogenic effects of resveratrol, various models were used to study its interaction with estrogen receptors. This can be referred to as another resveratrol paradox, in addition to the “French Paradox” [48,49], the “Estrogenic Paradox” of resveratrol has emerged, as can be determined by the fact that resveratrol is referred to as an estrogen receptor agonist [10,12], partial agonist [7,14], mixed agonist/antagonist [45] superagonist [8,10,46] or antagonist [50]. What should be emphasized is that the obtained results were often surprising and, without a doubt, dependent on the models used. This led to pioneering work by Gehm and coworkers [10], where estrogen-receptor-rich cytosolic extracts prepared from human MCF-7 breast cancer cells were used. The results indicated agonistic activity of resveratrol; however, resveratrol had a much lower affinity for the ERα than natural estrogens. Ashby and coworkers extended this research to include the use of rat uterine cytosolic extracts that express both ER subtypes and not just ERα [7]. In this case, the estimated IC_50_ for resveratrol was approximately five orders greater than the IC_50_ values for E2. Other groups performed similar binding assays on other cell models and reported similar results. For example, micromolar concentrations of resveratrol were required to displace nanomolar levels of E2 in cell extracts prepared from ER-expressing PR1 immortalized pituitary gland cells [51]. What is interesting is, in this study, resveratrol had a significantly weaker affinity for the ER than other phytoestrogens such as zearalenone, coumestrol and genistein [51]. Further studies in this area [35,45] showed that resveratrol has an affinity comparable to both ER isoforms or slightly higher than the ERβ isoform but confirmed that it is several orders of magnitude smaller than natural estrogens [35,45]. With the development of molecular biology methods, ER transactivation after incubation with resveratrol was studied in genetically modified yeast and mammalian cells [3,45]. In these experiments, luciferase genes were used as reporters [3,45]. The effect of resveratrol on ER transactivation in these models was inconsistent and depended on the model used. As it was mentioned before in the experiments employing MCF-7 cells transfected with luciferase reporter gene resveratrol-stimulated luciferase expression with an EC50 in the 5–10 µM range, and the effect was abolished by estrogen response element (ERE) deletion or treatment with an ER antagonist [10]. Moreover, these results indicated that resveratrol directly activated the ER. Surprisingly, it induced two to three times stronger the activity of the reporter gene than E2. Researchers have suggested that resveratrol may act as a superagonist. However, in the same study, expression of the reporter gene in transfected human BG-1 ovarian cancer cells after treatment with resveratrol was significantly lower than that of E2. The discrepancy in the results between MCF-7 and BG-1 cells suggests that the cellular environment modulates the agonistic activity of resveratrol. This may be modified by the expression of ER subtypes and the balance between coactivators and corepressors [10]. The superagonistic activity of resveratrol in MCF-7 cells was further described by two other groups [8,47] and in a subsequent study on MDA-MB-231 cells stably transfected with wild-type ERα [46]. Many studies, however, were published later as works assessing the estrogenic activity of resveratrol; the superagonistic activity of resveratrol in MCF-7 cells has not been reported anywhere. For instance, in the study performed in COS-1 monkey kidney fibroblast cells transfected with ERα or ERβ [7], resveratrol was significantly less potent than E2 and diethylstilbestrol on each receptor subtype, resulting in weaker responses. It was concluded that resveratrol with diethylstilbestrol was less effective against E2 and acted as an ER partial agonist at best [7]. The results of this study were confirmed in two studies employing CHO-K1 hamster ovary cells transiently transfected with ERα or ERβ. Resveratrol acted as an agonist that preferentially activated ERβ [45,52]. These results suggest that tissues in which ERβ are more highly expressed respond more to stimulation with resveratrol. The dependence of the estrogenic response of the environment in the cell was also suggested by experiments with Ishikawa endometrial adenocarcinoma cells conducted by Bhat and Pezzuto [35]. Resveratrol suppressed in a dose-dependent manner induced E2 expression of alkaline phosphatase (IC_50_ 2.3 µM) and progesterone receptor (PR) mRNA levels (range 5–15 µM) in human endometrial adenocarcinoma cells (Ishikawa). The decrease in PR expression was associated with a reduction in its cellular function, as evidenced by a decrease in the expression of integrin a1, a collagen-laminin receptor protein that is hormonally regulated in the endometrium. These studies led to the conclusion that resveratrol acted as an antiestrogen. Summarizing the results of these and several other studies, we face a scientific dilemma. There is disagreement as to whether resveratrol acts as a partial or full agonist of ER, as an antagonist or a superagonist. The results of our research presented in this paper provide new information on the structure dependence of estrogenic activity, suggesting the possibility of using resveratrol analogues as valuable tools in unraveling the following paradox next to the French Paradox related to resveratrol. Our results suggest that tested resveratrol analogues may act as agonists or antagonists of estrogen receptors. They may affect the proliferation of estrogen-dependent cancer cells, and they potentially regulate the expression of genes controlled by ERα and ERβ. However, this effect will be different in cells that vary in Erα, and ERβ expression might depend on the presence of E2 and can be modified by several other factors, e.g., expression of drug-metabolizing enzymes or transmembrane transporters [53,54].

Moreover, at higher concentrations of the tested compounds (1–10 µM), their cytostatic or cytotoxic effects unrelated to estrogen receptor modulation may play a leading role in their anticancer activity via, e.g., the induction of apoptosis or autophagy [1,2,3,4,11,55,56,57]. However, further studies are needed to clarify their mechanism of action.

## 3. Materials and Methods

### 3.1. Chemicals and Reagents

As shown in Table 1, resveratrol and its analogues were synthesized as described previously [58]. For this purpose, chemicals ordered from Sigma-Aldrich (St. Louis, MO, USA) and Alfa-Aesar (Ward Hill, MA, USA) were used. All cell culture media, supplements and compounds used for cell cultures were obtained from Sigma-Aldrich Co. (St. Louis, MO, USA).

### 3.2. Ligand-Binding Studies by Fluorescence Polarization

Binding affinities to ERα and ERβ were determined by the dose dependence binding of the test compounds with purified human Erα and Erβ. All chemicals were obtained from Panvera (Göttingen, Germany). The assay was performed according to the protocols recommended by the manufacturer. Compounds were dissolved in DMSO as control DMSO alone was used and as positive controls 17β-estradiol (agonist) and tamoxifen (antagonist) were used. Increasing concentrations of ligands (tested compound) were added to estrogen receptor (ERα or ERβ) and a fluorescent peptide (D22), which resulted in either the formation or disruption of the ER/D22 complex. D22 is a coactivator-like peptide containing an LXXLL motif and flanking sequences that resemble known coactivators. Agonist-bound ER (ERα or ERβ) can recruit D22, resulting in a more significant fraction of bound D22 and a larger polarization value. Antagonist-bound ER has a lower affinity for D22, yielding a more significant fraction of unbound D22 and a lower polarization value. Recombinant human ERα or ERβ were incubated with the tested compounds for one hour. Fluorescence polarization was measured using a Tecan Ultra Evolution multiwell plate reader (Tecan, Crailsheim, Germany) at excitation wavelength 540 nm and emission wavelength 580 nm. The concentration of the ligand that resulted in a half-maximum increase or decrease in polarization was equal to ligand EC_50_ for the ERα and ERβ interaction. The EC_50_ values were derived by logarithmic curve-fitting from binding curves using GraphPad Prism 8.4.3. GraphPad Software, San Diego, California, USA. EC_50_ was determined from at least three independent experiments. The EC_50_ values of the ligands, which either promote or disrupt the ER/D22 interaction, provided a means to classify the test compound as an antagonist, agonist or selective modulator.

### 3.3. Computational Details

The optimization of the ligands (Table 1) using the Gaussian 16 C.01 program [59] and density functional theory (DFT) formalism with the B3LYP/6-31G(d,p) (very tight criteria) [60] approximation. The crystal structure of the human ERα LBD (PDB entries: 3dt3 or 1l2j.pdb for ERα or ERβ, respectively) [26,27] with the resolution 2.400 Å (for ERα) or 2.950 Å (for ERβ) was selected as the biological target as one of the most used for docking PDB versions of the human estrogen receptor ERα and ERβ. To carry out docking simulations (using the AutoDock Vina package [61]), a grid box was defined to be of 10 Å size (ERα: center_x = 41.526, center_y = 1.476, center_z = 15.981; ERβ: center _x = 31.926, center_y = 82.682, center_z = −11.054). The outputs (Figure 2) after the docking procedure (the projections of the 1st poses) were visualized using the LigPlot + v.2.2 EMBL-EBI, Cambridgeshire, UK [62,63]. The additional SAPT (symmetry-adapted perturbation theory) analysis of the ligand–amino acid complexes was performed. The interaction energy was estimated using Psi4 1.3.2 The Psi 4 Project [64], treating the complexes lig-and-amino acid as a closed-shell system and utilizing the recommended jun-cc-pVDZ basis set. The detailed protocol is described in our previous studies [30].

### 3.4. Cell Culture

Cell lines were obtained from ETCC. MCF-7 estrogen-dependent breast cancer cells were maintained in DMEM, while the Ishikawa cell line was kept in DMEM/F12. The cell culture media was supplemented with 10% fetal bovine serum, 1% antibiotics (penicillin and streptomycin) and L-glutamine, without phenol red. Cell lines were cultured in an incubator at 37 °C and in an atmosphere of 5% CO_2_ and 95% air. For the experiments, FBS has been replaced by its charcoal-treated equivalent.

### 3.5. Ishikawa Cells Proliferation

The estrogen-stimulated proliferation of Ishikawa cells was performed according to Wober and coworkers [36] with some modifications: Ishikawa cells were seeded at a density of 5 × 10^3^ cells/well in 96-well plates in phenol red-free DMEM/F12 containing 10% charcoal dextran-treated FBS. After 72 h, cells were washed with PBS and kept in the same media for the next 24 h. Then, the medium was changed, and cells were incubated with the tested compounds at concentrations ranging from 10^1^ to 10^4^ nM for 72 h. As a positive control, E2 at a concentration of 1 nM was used. For the competition assay, a range of concentrations of resveratrol analogues was added concurrently with 1 nM estradiol. The treated cells were grown for 72 h. The estrogenic stimulation was evaluated by alkaline phosphatase activity. After incubation, the cells were washed with PBS, and the plates were frozen at −20 °C until analysis. After thawing, the plates were warmed in a water bath up to 22 °C and incubated with the p-Nitrophenyl Phosphate Liquid Substrate System (Sigma-Aldrich). This ready-to-use, single-solution reagent contains p-nitrophenyl phosphate, buffer and the necessary magnesium cations. Alkaline phosphatase releases p-nitrophenol, measured kinetically at 405 nm using plate reader Biotek Instruments, Elx-800, Winooski, VT, USA).

### 3.6. MCF-7 Cells Proliferation

The estrogen-stimulated proliferation of MCF-7 cells was performed using Hoechst 33258 DNA staining [65]. MCF-7 cells were seeded at a density of 1.5 × 10^4^ cells/well in 24-well plates in phenol red-free DMEM containing 5% charcoal dextran-treated FBS. After 24 h, cells were washed with PBS and kept in the same media for the next 48 h. Then, cells were incubated with tested compounds at concentrations ranging from 10^1^ to 10^4^ nM for 96 h. E2 at 1 nM and 10 nM was used as a positive control. In the estrogen receptor competition assay, a range of concentrations of resveratrol analogues was added together with 1 nM estradiol. Proliferation was assessed by the fluorometric measurement of DNA. For this purpose, cells were washed with PBS, lysed using EDTA (0.5 mL, 10 min, pH 12.3) at 37 °C for 30 min and neutralized with 10 mM KH_2_PO_4_, and then, Hoechst 33258 (1 mg/mL) was added. Excitation at 350 nm and emission at 455 nm was used for fluorescence measurement using Tecan Infinite M Plex microplate reader (Männedorf, Switzerland) [65].

### 3.7. Statistical Analysis

Statistical analyses were carried out using one-way ANOVA with Dunnett’s multiple comparison tests. The results were presented as the mean ± SD from three independent experiments. The values were calculated using GraphPad Prism version 8.00 for Windows (GraphPad Software, San Diego, CA, USA).

## 4. Conclusions

Of 12 tested compounds, two (resveratrol and **M8**) showed agonistic activity towards Erα and Erβ. At the same time, two analogues: penta- and hexahydroxy stilbenes (**M11** and **M12**) showed antagonistic activity towards the ERα receptor. The agonistic activity of resveratrol and **M8** on ERα can also be confirmed from data obtained by in silico docking and SAR analysis. These findings originate from the data covering the interactions of ligands with Trp383 and Glu353 (within the cavity of the 3dt3.pdb protein). On the other hand, the ERβ ligands **M8**, **M11** and **M12** seemed to be more effective agonists. The resulting binding modes for **M8**, **M11** and **M12** were similar. Moreover, in most cases, more potent interactions with amino acids within the cavity of the 1l2j.pdb protein (ERβ) were responsible for the mentioned alkenes. In in vitro studies, resveratrol analogues at concentrations of 10–100 nM stimulated the proliferation of estrogen-dependent Ishikawa and MCF-7 cells. In contrast, at concentrations of 100–1000 nM, they were cytostatic or cytotoxic, indicating other factors’ influence on the proliferation of Ishikawa and MCF-7 cells. The exception was resveratrol, which stimulated the proliferation of these cells in concentrations of 100–1000 nM. Most of the tested compounds incubated with tested cells in the presence of 1nM estradiol modulated its effect on the proliferation, which proves there is a competition for access to the estrogen receptor. Therefore, our results indicate that resveratrol and its derivatives might exert agonistic and antagonistic activity toward estrogen receptors. Moreover, the expression of genes regulated by ERs and the proliferation of estrogen-dependent cancer cells might change due to resveratrol derivatives treatment. It should be highlighted that the dose-dependent activity of resveratrol derivatives may translate into an antitumor effect in vivo. Since the agonistic activity of analogues can promote cancer cell proliferation at lower doses, they should be administered cautiously to avoid cancer progression. On the other hand, as we observed in our study, the cytostatic or cytotoxic effects can occur independently from estrogen receptor modulation at higher doses. Moreover, this duality of action is crucial when the bioavailability of these compounds is low, and the obtained blood concentrations will be predisposed toward ER modulating activity rather than affect cancer cell growth. However, further detailed studies are needed to clarify the activity of tested compounds and determine their anticancer potential.

## Figures and Tables

**Table 1:** The structure of the tested compounds **M 1–12**, resveratrol, possesses the number 7 according to the code used in the table below.

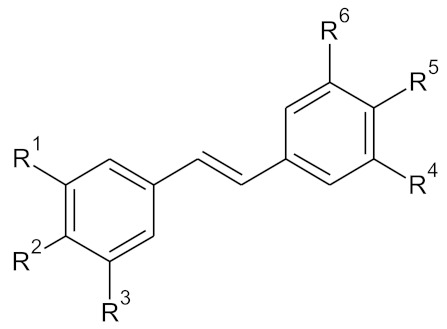						
Compound	Pos. 3 (=R^1^)	Pos. 4 (=R^2^)	Pos. 5 (=R^3^)	Pos. 3′ (=R^4^)	Pos. 4′ (=R^5^)	Pos. 5′ (=R^6^)
**M1**	-OCH₃	-H	-OCH₃	-H	-OCH₃	-H
**M2**	-OCH₃	-OCH₃	-OCH₃	-H	-OCH₃	-H
**M3**	-OCH₃	-H	-OCH₃	-OCH₃	-H	-OCH₃
**M4**	-OCH₃	-H	-OCH₃	-OCH₃	-OCH₃	-H
**M5**	-OCH₃	-OCH₃	-OCH₃	-OCH₃	-H	-OCH₃
**M6**	-OCH₃	-OCH₃	-OCH₃	-OCH₃	-OCH₃	-OCH₃
**M7**	-OH	-H	-OH	-H	-OH	-H
**M8**	-OH	-OH	-OH	-H	-OH	-H
**M9**	-OH	-H	-OH	-OH	-H	-OH
**M10**	-OH	-H	-OH	-OH	-OH	-H
**M11**	-OH	-OH	-OH	-OH	-H	-OH
**M12**	-OH	-OH	-OH	-OH	-OH	-OH

**Table 2:** Interaction of resveratrol and its analogues with estrogen receptors—EC_50_ values.

Compound	ERα Agonist EC_50_ (nM)	ERβ Agonist EC_50_ (nM)
resveratrol	21.2 ± 2.2	32.3 ± 3.6
**M8**	108.5 ± 10.2	8.1 ± 1.6
estradiol	8.3 ± 2.1	3.1 ± 0.9
**Compound**	**ERα antagonist EC_50_ (nM)**	**ERβ antagonist EC_50_ (nM)**
**M11**	1012.3 ± 30.5	>5000
**M12**	110.2 ± 15.5	>5000
tamoxifen	25.1 ± 3.6	240.2 ± 14.5

**Table 3:** ERα calculated total values of the interaction ligand amino acid energy (kcal/mol) using the SAPT0 method for docked ligands.

Contacts	Electrostatics	Exchange	Induction	Dispersion	Total SAPT0
Glu353_estradiol	−16.48923	20.23436	−8.87314	−4.35977	−15.11974
Glu353_**M11**	−5.58802	8.24529	−1.55134	−6.79997	−9.07403
Glu353_**M12**	−7.23545	7.55212	−1.67306	−7.19391	−13.62578
Glu353_**M8**	−3.10338	28.25146	−9.06124	−6.54009	15.21372
Glu353_resveratrol	−0.04736	7.90314	−1.92755	−3.18726	4.36802
Glu353_tamoxifen	0.06031	0.00046	−0.02167	−0.24192	−0.32322
Leu346_estradiol	−2.98294	4.04051	−0.88196	−4.95162	−7.61105
Leu346_**M11**	−0.12331	0	−0.00235	−0.01384	−0.22231
Leu346_**M12**	0.17741	0	−0.00204	−0.01417	0.25688
Leu346_**M8**	1.61081	0.8349	−0.41181	−2.44566	−0.65619
Leu346_resveratrol	−0.69714	0.04584	−0.11532	−1.24237	−3.20152
Leu346_tamoxifen	−0.09257	0.00032	−0.01715	−0.16193	−0.43239
Leu387_estradiol	−7.32042	27.15115	−4.08131	−11.49336	6.78245
Leu387_**M11**	−1.97959	8.37737	−2.2794	−5.13834	−1.62543
Leu387_**M12**	−1.93474	7.15897	−1.66628	−4.70677	−1.83076
Leu387_**M8**	0.70104	2.78983	−0.66255	−4.62453	−2.86244
Leu387_resveratrol	−4.72523	14.06595	−2.517	−9.62002	−4.45619
Leu387_tamoxifen	−0.0311	0.03712	−0.00821	−0.37893	−0.60736
Phe404_estradiol	−5.86981	26.08135	−3.4992	−12.04759	7.43375
Phe404_**M11**	−0.06108	−0.00002	−0.01645	−0.09533	−0.2755
Phe404_**M12**	0.0765	−0.00002	−0.01385	−0.09806	−0.05646
Phe404_**M8**	−1.37589	3.62802	−0.65048	−5.83775	−6.75066
Phe404_resveratrol	−2.67406	10.12713	−1.38583	−7.92448	−2.9597
Phe404_tamoxifen	−0.01185	0	−0.00042	−0.01658	−0.04596
Trp383_estradiol	−0.08804	0.02432	−0.02605	−0.6719	−1.21379
Trp383_**M11**	−0.6325	0.00003	−0.03516	−0.12425	−1.26194
Trp383_**M12**	0.51117	0.00002	−0.0319	−0.12425	0.5658
Trp383_**M8**	−0.29856	0.00114	−0.02108	−0.44586	−1.21807
Trp383_resveratrol	−0.30921	0.00078	−0.02223	−0.31586	−1.0303
Trp383_tamoxifen	−3.32377	11.98675	−1.40812	−10.75084	−5.57119

**Table 4:** ERβ Calculated total values of the interaction ligand amino acid energy (kcal/mol) using the SAPT0 method for docked ligands.

Contacts	Electrostatics	Exchange	Induction	Dispersion	Total SAPT0
Gly472_estradiol	0.67436	2.07195	−0.42113	−2.26001	0.10386
Gly472_**M11**	−0.35218	0.93438	−0.26853	−1.18058	−1.38151
Gly472_**M12**	1.32746	0.85342	−0.27736	−1.22162	1.08668
Gly472_**M8**	2.18831	4.18735	−0.84722	−2.52704	4.78306
Gly472_resveratrol	1.18342	3.43909	−0.56283	−2.29321	2.81503
Gly472_tamoxifen	−0.00946	0	−0.00005	−0.00121	−0.01708
His475_estradiol	2.6531	0.32233	−0.2649	−1.1753	2.44654
His475_**M11**	−8.82514	7.30577	−2.10095	−3.77149	−11.7796
His475_**M12**	−9.74329	16.46084	−4.11932	−6.04703	−5.49602
His475_**M8**	−5.37217	7.33722	−1.4282	−4.28748	−5.97701
His475_resveratrol	−7.87121	7.70495	−1.86333	−3.38573	−8.62985
His475_tamoxifen	0.00019	0	0	−0.00103	−0.00132
Ile376_estradiol	−1.30063	6.40377	−1.14341	−3.91051	0.07843
Ile376_**M11**	−0.79526	2.10206	−0.40792	−2.30483	−2.24053
Ile376_**M12**	−0.01665	1.65675	−0.38003	−2.25141	−1.57979
Ile376_**M8**	−0.19615	1.61684	−0.22421	−2.36771	−1.86646
Ile376_resveratrol	−0.62198	4.52934	−0.69196	−3.92697	−1.13396
Ile376_tamoxifen	0.02073	0	−0.00017	−0.00308	0.02785
Ile380_estradiol	0.03227	0.01127	−0.00866	−0.50132	−0.74332
Ile380_**M11**	0.08539	0.00143	−0.00339	−0.22014	−0.21785
Ile380_**M12**	0.0868	0.00136	−0.00482	−0.2241	−0.22432
Ile380_**M8**	0.04291	0.0004	−0.00576	−0.21957	−0.29007
Ile380_resveratrol	0.13932	0.00055	−0.0065	−0.24107	−0.17163
Ile380_tamoxifen	0.01516	0	−0.00034	−0.01263	0.00349
Leu298_estradiol	−0.04451	0.23119	−0.12607	−1.75834	−2.70551
Leu298_**M11**	−3.442	0.51532	−0.49191	−2.31155	−9.13157
Leu298_**M12**	0.86562	0.34692	−0.21914	−2.18667	−1.90159
Leu298_**M8**	0.57419	1.13771	−0.37985	−2.36568	−1.64719
Leu298_resveratrol	−3.26084	2.76471	−0.95337	−2.90107	−6.93308
Leu298_tamoxifen	−0.03978	0	−0.00095	−0.01014	−0.08108
Leu339_estradiol	−3.67128	8.14513	−1.27868	−9.21987	−9.60098
Leu339_**M11**	−1.31019	8.0409	−1.70312	−7.86206	−4.51701
Leu339_**M12**	−6.77811	7.65731	−1.62827	−7.81049	−13.6405
Leu339_**M8**	−2.9227	5.78307	−0.93685	−6.63826	−7.51341
Leu339_resveratrol	−1.34789	7.12232	−1.18906	−7.29872	−4.324
Leu339_tamoxifen	0.21284	0.14239	−0.0332	−0.84432	−0.83233
Leu343_estradiol	−2.18674	13.09303	−2.21457	−6.18217	3.99922
Leu343_**M11**	−2.36232	5.97714	−0.90422	−3.47968	−1.22563
Leu343_**M12**	−1.89879	6.0387	−0.83481	−3.65332	−0.55493
Leu343_**M8**	−3.74278	7.31482	−1.18679	−3.67933	−2.06224
Leu343_resveratrol	0.54534	8.09066	−2.46044	−4.20701	3.13709
Leu343_tamoxifen	0.06469	0.21826	−0.10876	−1.02673	−1.3586
Met336_estradiol	−4.83937	13.30994	−1.95439	−7.8959	−2.19874
Met336_**M11**	−0.87805	0.07175	−0.13742	−1.25052	−3.49673
Met336_**M12**	−0.3888	0.08958	−0.13754	−1.31122	−2.78558
Met336_**M8**	−0.61499	2.07571	−0.46877	−3.18665	−3.49747
Met336_resveratrol	−0.62168	1.34148	−0.35293	−2.64949	−3.6376
Met336_tamoxifen	0.00693	−0.00001	−0.00365	−0.03468	−0.05005
Met340_estradiol	−0.00016	0	0.00001	0	−0.00023
Met340_**M11**	−0.93804	8.09994	−1.91689	−3.86824	2.19404
Met340_**M12**	−1.22265	6.13644	−1.08073	−3.37871	0.72404
Met340_**M8**	−1.21279	3.35209	−0.42284	−2.76341	−1.66842
Met340_resveratrol	−1.47083	2.07677	−0.34852	−2.7766	−4.01457
Met340_tamoxifen	0.23072	0.00275	−0.00888	−0.23187	−0.0116

## Data Availability

Not applicable.

## Sample Availability

Samples of the compounds are available from the authors.
